# Genetic Markers Enhance Coronary Risk Prediction in Men: The MORGAM Prospective Cohorts

**DOI:** 10.1371/journal.pone.0040922

**Published:** 2012-07-25

**Authors:** Maria F. Hughes, Olli Saarela, Jan Stritzke, Frank Kee, Kaisa Silander, Norman Klopp, Jukka Kontto, Juha Karvanen, Christina Willenborg, Veikko Salomaa, Jarmo Virtamo, Phillippe Amouyel, Dominique Arveiler, Jean Ferrières, Per-Gunner Wiklund, Jens Baumert, Barbara Thorand, Patrick Diemert, David-Alexandre Trégouët, Christian Hengstenberg, Annette Peters, Alun Evans, Wolfgang Koenig, Jeanette Erdmann, Nilesh J. Samani, Kari Kuulasmaa, Heribert Schunkert

**Affiliations:** 1 UKCRC Centre of Excellence for Public Health, Queens University of Belfast, Belfast, Northern Ireland, United Kingdom; 2 Department of Chronic Disease Prevention, National Institute for Health and Welfare, Helsinki, Finland; 3 Universität zu Lübeck, Lübeck, Germany; 4 FIMM, Institute for Molecular Medicine Finland, University of Helsinki, Helsinki, Finland; 5 Institute of Epidemiology II, Helmholtz Zentrum München, München, Germany; 6 INSERM U744, Lille, France; 7 Department of Epidemiology and Public Health, EA3430, University of Strasbourg, Strasbourg, France; 8 INSERM U558, Toulouse University School of Medicine, Toulouse, France; 9 Department of Public Health and Clinical Medicine, Umeå University, Umeå, Sweden; 10 INSERM UMRS937, University Pierre et Marie Currie, Paris, France; 11 Department of Internal Medicine and Cardiology, University of Regensburg, Regensburg, Germany; 12 Department of Internal Medicine II–Cardiology, University of Ulm, Ulm, Germany; 13 Department of Cardiovascular Sciences, University of Leicester, and Leicester NIHR Biomedical Research Unit in Cardiovascular Disease, Glenfield Hospital, Leicester, United Kingdom; Medizinische Hochschule Hannover, Germany

## Abstract

**Background:**

More accurate coronary heart disease (CHD) prediction, specifically in middle-aged men, is needed to reduce the burden of disease more effectively. We hypothesised that a multilocus genetic risk score could refine CHD prediction beyond classic risk scores and obtain more precise risk estimates using a prospective cohort design.

**Methods:**

Using data from nine prospective European cohorts, including 26,221 men, we selected in a case-cohort setting 4,818 healthy men at baseline, and used Cox proportional hazards models to examine associations between CHD and risk scores based on genetic variants representing 13 genomic regions. Over follow-up (range: 5–18 years), 1,736 incident CHD events occurred. Genetic risk scores were validated in men with at least 10 years of follow-up (632 cases, 1361 non-cases). Genetic risk score 1 (GRS1) combined 11 SNPs and two haplotypes, with effect estimates from previous genome-wide association studies. GRS2 combined 11 SNPs plus 4 SNPs from the haplotypes with coefficients estimated from these prospective cohorts using 10-fold cross-validation. Scores were added to a model adjusted for classic risk factors comprising the Framingham risk score and 10-year risks were derived.

**Results:**

Both scores improved net reclassification (NRI) over the Framingham score (7.5%, *p* = 0.017 for GRS1, 6.5%, *p* = 0.044 for GRS2) but GRS2 also improved discrimination (c-index improvement 1.11%, *p* = 0.048). Subgroup analysis on men aged 50–59 (436 cases, 603 non-cases) improved net reclassification for GRS1 (13.8%) and GRS2 (12.5%). Net reclassification improvement remained significant for both scores when family history of CHD was added to the baseline model for this male subgroup improving prediction of early onset CHD events.

**Conclusions:**

Genetic risk scores add precision to risk estimates for CHD and improve prediction beyond classic risk factors, particularly for middle aged men.

## Introduction

Coronary heart disease is a leading cause of morbidity and mortality among adults in Western societies [1]. Both lifestyle and genetic factors contribute to the manifestation of the disease. Current risk scores, based on age, sex and modifiable risk factors such as blood lipid profile explain a significant proportion of coronary heart disease (CHD) [2] Pharmacologic preventive therapies are aimed at those at high risk (>20% 10-year risk of CHD). However, a substantial population attributable fraction of CHD comes from those at intermediate risk (i.e. 5–<20% 10 years CHD risk) and 15–20% of myocardial infarction (MI) patients would be considered low risk using current risk scores [2]. Particularly, men and individuals with a positive family history of coronary heart disease carry a high lifetime risk [1]. More accurate prediction, specifically in middle-aged men, is needed to reduce the burden of disease more effectively.

Genome wide association (GWA) studies have identified several common genetic variants associated with modest population attributable fractions for CHD [3]. Prediction improvement using genetic markers must be demonstrated over and above well-validated risk scores using standard metrics to evaluate their performance including discrimination, calibration, risk reclassification and, thereby, their potential clinical utility [4]–[5]. The most validated genetic risk marker for CHD is on chromosome 9p21.3 [6]. It's individual utility in CHD prediction is modest [6]. Combining the relatively small effects of individual variants into a multilocus genetic risk score (GRS) may improve prediction and thereby clinical decision-making for primary prevention. However, recent efforts produced rather mixed results [7]–[9]. For example, a GRS combining the effects of 101 SNPs failed to improve prediction beyond family history in a large cohort of women [9]. However, most of these SNPs could not be validated in large scale GWAS to be significantly associated with CHD, until recently 13 loci [10] were reproducibly associated with CHD but 16 new loci [11]–[12] have been added to this group. Other studies have tested the value of scores based on risk alleles from smaller subsets of SNPs, which have improved prediction in case-control groups, but the results may not generalise to the prospective setting [7]. Indeed, a 13 SNP GRS weighted with effect size estimates from previous GWA studies failed to substantially improve CHD prediction in prospective cohorts from Sweden and Finland [8] or America [13]. Similarly a 29 SNP score provided only marginal predictive benefit in a prospective Dutch cohort but this effect was mainly contributed by three SNPs [14].

In this study, we devised a multilocus GRS for CHD prediction, combining variants from 13 genomic regions, in the prospective MORGAM (MOnica Risk, Genetics, Archiving, Monograph) cohorts [15]. We tested the performance of GRS using both published effect estimates from GWA studies and estimates derived from the MORGAM cohorts. Compared with previous studies, our analysis is based on a larger number of incident CHD events from a wider selection of European populations with specific focus on middle aged men.

## Methods

### Study population

**Figure 1:**
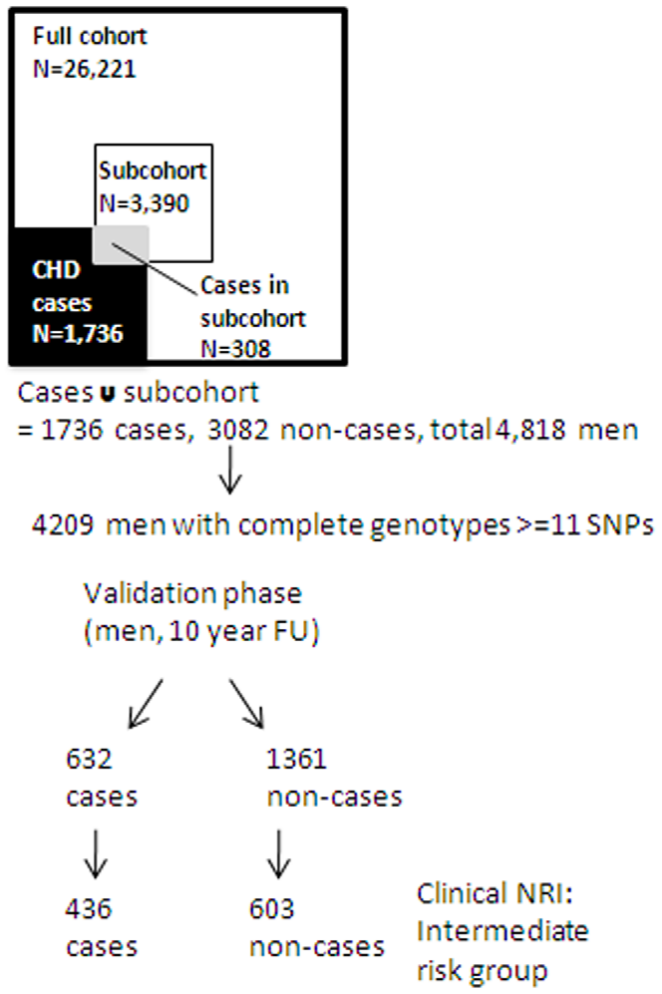
Schematic diagram of the case-cohort design in MORGAM. Outline of the selection of individuals in the MORGAM dataset. The subcohort and all CHD cases who were genotyped (N = 4818) were chosen from the full cohort. We restricted the validation analysis to 4209 men with complete genotype data for > = 11 SNPs with the remaining SNP data multiply imputed.

The MORGAM project comprises >128,000 men and women from 57 European (mainly white Caucasian) cohorts which were harmonised and prospectively followed up for incident coronary heart disease events [15]. For this analysis we focused on 26,221 men without MI at baseline from nine cohorts. Of these, 1736 men developed incident CHD (fatal and non-fatal) over a median 18 years follow up. From the full eligible cohort, a random subcohort was selected independently of the case selection, with selection probabilities depending on age. The case-cohort set comprises the subcohort along with all cases outside the subcohort, resulting in a total of 4818 men (1736 cases and 3082 non-cases) for genotyping (Table 1, Figure 1) [16]. Baseline characteristics were similar across cohorts (Table 1, Table S1).

**Table 1:** Background characteristics of the nine cohorts.

	FINRISK Eastern Finland	FINRISK South western Finland	ATBC, Southern Finland	PRIME Lille, France	PRIME Toulouse France	PRIME Strasbourg France	PRIME Belfast UK	MONICA KORA Augsburg Germany	Northern Sweden
*Baseline characteristics*									
Total number	3391	2817	4565	2358	2413	2276	2543	4505	1353
Age (years)	46.5 (12.5)	47.3 (13.0)	62.7 (4.9)	55.2 (2.9)	54.9 (2.8)	54.7 (2.9)	54.8 (2.9)	53.4 (10.8)	45.2 (11.2)
Cholesterol									
Total, mmol/L	5.64 (1.08)	5.53 (1.08)	5.94 (1.03)	5.74 (0.96)	5.52 (0.89)	5.81 (0.99)	5.88 (1.02)	6.21 (1.18)	6.22 (1.25)
HDL mmol/L	1.27 (0.32)	1.25 (0.31)	1.18 (0.31)	1.34 (0.33)	1.25 (0.30)	1.26 (0.34)	1.19 (0.32)	1.29 (0.39)	1.26 (0.31)
Ratio of total to HDL	4.71 (1.52)	4.67 (1.49)	5.40 (1.89)	4.57 (1.50)	4.68 (1.41)	4.91 (1.60)	5.27 (1.58)	5.34 (5.15)	5.24 (1.65)
Blood pressure									
Systolic (mm Hg)	139.6 (18.8)	138.3 (18.0)	141.5 (18.3)	138.7 (19.1)	126.6 (14.8)	135.0 (18.3)	134.0 (20.4)	138.3 (18.6)	129.6 (16.9)
Diastolic (mm Hg)	83.8 (11.8)	84.9 (11.6)	84.9 (10.1)	86.5 (12.2)	79.5 (9.8)	86.7 (11.1)	81.9 (11.5)	83.9 (11.5)	82.6 (11.2)
BMI (kg/m^2^)	27.0 (4.0)	26.7 (3.8)	26.8 (4.1)	26.5 (3.5)	26.3 (3.2)	27.4 (3.5)	26.2 (3.4)	27.4 (3.5)	25.9 (3.5)
Daily smoker	979 (28.9)	849 (30.1)	3569 (78.2)	429 (18.2)	443 (18.4)	383 (16.8)	591 (23.2)	1206 (26.8)	277 (20.5)
Current drug therapy									
Lipid lowering	83 (2.4)	68 (2.4)	NA	310 (13.1)	330 (13.7)	228 (10.0)	30 (1.2)	NA	8 (0.6)
High BP medication	424 (12.5)	293 (10.4)	NA	365 (15.5)	328 (13.6)	283 (12.4)	223 (8.8)	524 (11.6)	78 (5.8)
History of diabetes	168 (5.0)	109 (3.9)	233 (5.1)	127 (5.4)	106 (4.4)	100 (4.4)	48 (1.9)	237 (5.3)	30 (2.2)
History of hypertension	1188 (35.0)	897 (31.8)	NA	586 (24.9)	596 (24.7)	505 (22.2)	397 (15.6)	1375 (30.5)	224 (16.6)
Prevalent cases N, %									
stroke	75 (2.2)	58 (2.1)	194 (4.2)	18 (0.8)	9 (0.4)	15 (0.7)	15 (0.6)	78 (1.7)	17 (1.3)
Family history of CHD	924 (27.2)	531 (18.8)	NA	205 (8.7)	176 (7.3)	136 (6.0)	449 (17.7)	801 (17.8)	132 (9.8)
Person years of Follow-up	C3 = 16,160	C3 = 17,381	29158	22,766	22,518	21,898	12,333	C1 = 19,468	C2 = 5413
	C24 = 17,878	C24 = 12,436						C2 = 16,829	C3 = 3829
								C3 = 10,836	
*Incident CHD events during follow-up N, %*									
men	254 (7.5)	184 (6.5)	436 (9.6)	137 (5.8)	145 (6.0)	131 (5.8)	106 (4.2)	304 (6.7)	39 (2.9)
*Random subsample of the case cohort set*									
men	404 (11.9)	298 (10.6)	961 (21.1)	116 (4.9)	121 (5.0)	125 (5.5)	160 (6.3)	1121 (24.9)	84 (6.2)

Data are mean (SD) or number %. NA = not available, C = cohort. As ATBC and MONICA-KORA did not collect information on current drug therapy, these were considered as ‘no medication’ for the analysis.

We focused our analysis on men as this approach neutralizes the dominant effect of gender on CHD prediction, which marginalizes other variables in standard risk models while providing clinically relevant information specific to men. The cohorts were measured at baseline in a highly standardised way for total and high-density lipoprotein (HDL) cholesterol, and blood pressure. Questionnaire data were collected on daily smoking, history of MI at baseline, history of diabetes blood pressure medication use and family history of CHD [17]. The follow-up procedures of the cohorts varied, depending on what was possible to do in each country: linkage to hospital discharge and mortality registries, linkage to WHO MONICA coronary event registries or active follow-up through re-contact to the cohort participants. Possible coronary events were validated using medical records for clinical symptoms and signs, ECG reports, cardiac biomarkers and enzymes, and death certificates and autopsy reports. In some of the cohorts, where validation studies had shown good agreement, all or some of the end-points were classified on the basis of the clinical and/or routine death diagnoses. Incident CHD was defined as first fatal or non-fatal CHD event, which included definite and possible acute MI or coronary death, unstable angina pectoris, revascularization and unclassifiable fatal events [17]. Details of the follow-up and diagnostic procedures in each cohort have been published [18].

### SNP selection and genotyping

We chose SNPs that exceeded a significance threshold of p<5×10^−8^ for association with CHD or MI and were replicated in (at least two) large GWAs studies. 12 SNPs and a haplotype comprising four SNPs from the LPA locus met these criteria at study initiation (see Table 2 for references). SNP genotyping was carried out on the MassARRAY System with iPLEX Gold chemistry (Sequenom, California) as previously described [22].

**Table 2:** Association between SNPs and coronary heart disease.

Locus	Candidate gene(s)	SNP	Risk Allele	Frequency	Other allele	orientation in MORGAM	HR reported in ref	Ref	Coronary heart disease (1736 cases, 3082 non cases) men only	
									Pooled HR (95% C.I.)	*p* value
1q41	MIA3	rs3008621	A	0.14	G*	FT	1/1.08	[10]	0.85 (0.72, 0.99)	0.045
1p32.3	PCSK9	rs11206510	C	0.17	T*	FB	1/1.15	[11]	1.01 (0.89, 1.16)	0.83
1p13.3	PSRC1	rs646776	A	0.78	G	FT	1.19	[19]	1.10 (0.98, 1.24)	0.11
3q22.3	MRAS	rs9818870	C	0.86	T	FB	1/1.15	[20]	0.99 (0.83, 1.19)	0.98
2q33.1	WDR12	rs6725887	C*	0.13	T	FB	1.14	[11]	1.04 (0.9, 1.2)	0.58
6q25.3	SLC22A3 H∧	rs2048327	A	0.66	G	FT		[21]	0.87 (0.79, 0.97)	0.01
6q25.3	LPAL2 H∧	rs3127599	A	0.32	G	FT		[21]	1.01 (0.91, 1.1)	0.88
6q25.3	LPA H∧	rs7767084	C	0.16	T	FB		[21]	1.11 (0.96, 1.23)	0.15
6q25.3	LPA H∧	rs10755578	C	0.54	G	FT		[21]	0.92 (0.83, 1.01)	0.10
6p24.1	PHACTR1	rs12526453	C*	0.68	G	FB	1.1	[11]	1.14 (1.02, 1.27)	0.017
9p21.3	ANRIL/CDKN2A/B	rs1333049	C*	0.46	G	FB	1.2	[10]	1.26 (1.14, 1.4)	0.00001
10q11.21	CXCL12/SDF1	rs501120	C	0.15	T*	RB	1/1.11	[10]	1.09 (0.92, 1.3)	0.30
12q24	SH2B3	rs3184504	C	0.54	T*	FB	1/1.07	[11]	1.03 (0.93, 1.14)	0.62
12q24.31	HNF1A/C12orf43	rs2259816	A*	0.36	G	FT	1.08	[20]	0.99 (0.9, 1.11)	0.97
19p13.2	LDLR	rs1122608	G*	0.77	T	FB	1.14	[11]	0.99 (0.88, 1.12)	0.95
21q22	SLC5A3/MRPS6	rs9982601	C	0.85	T*	FB	1/1.18	[11]	0.92 (0.80, 1.06)	0.26

In MORGAM the alphabetically first allele for each SNP was used as the explanatory variable (‘risk allele’) in this analysis. Orientation of alleles in MORGAM is given as FT forward top, FB forward bottom, RB reverse bottom. Logarithms of the odds ratios (ORs) from references were used as SNP coefficients in genetic risk score (GRS1). In the score the coefficient was placed on the MORGAM risk allele; when this was different from the risk allele reported in the literature (indicated by *), we used the log inverse (1/OR) of the reported OR as the coefficient. The rs9818870 SNP was not genotyped for the Swedish cohort due to technical difficulties. All SNPs except rs9818870 were included in GRS1/GRS2. H† indicates haplotypes derived from these SNPs. SNP associations were tested with a model adjusted for cohort and Framingham coefficients.

### Statistical analysis

Missing genetic data (which ranged from 10.2% to 21.4% (mean 13.1%) per SNP) were multiply imputed within each cohort using WinBUGS [23], with outcome information included in the imputation model to avoid attenuation of estimated effects in later analyses [24]. Multiple imputation is a statistical technique distinct from imputation of SNP information based on linkage disequilibrium. The main purpose of multiple imputation, rather than to estimate individual predictor values, is to estimate the uncertainty about the missing values, while at the same time minimising the information loss compared to complete case analysis (see Supporting Information S1). Because of the case-cohort design, all analyses used inverse selection probability weighting to relate the results back to the larger cohort level.

### Individual SNP/haplotype association analysis

We tested the association between individual SNPs/haplotypes and incident CHD events using Cox proportional hazards models adjusted for cohort and geographical region and a score of classic risk factors at baseline based on Framingham coefficient of age, daily smoking, systolic blood pressure, total cholesterol, HDL-cholesterol, and prevalent diabetes [25]. We refer to this as the Framingham Score. In the Framingham study these risk estimates gave a c-index of 0.742 for CHD [25] We applied these coefficients in the MORGAM data, but allowed the baseline risk to vary between cohorts (i.e. absolute cohort specific risk levels were estimated from MORGAM data), resulting in a c-index of 0.7428. The relative risk in terms of the Framingham score was higher in the ATBC cohort which is in part due to the larger percentage of smokers in this cohort (Figure S1). However, the genetic score is evenly distributed across the cohorts (Figure S2) and uncorrelated with the Framingham score, so the differences in cohort characteristics are unlikely to be a source of confounding in the genetic risk score analyses. For the LPA locus, we used FastPHASE to multiply impute LPA haplotype pairs separately for cases and non-cases within each cohort [26]. We estimated the effect sizes from the LPA haplotypes using a model that simultaneously included all haplotypes compared to the most frequent AATC haplotype [21]. In the Cox regression models all missing data were handled using multiple imputation. However, in validation of the prediction models we limited the amount of missing data by restricting the validation set to men with complete genotypes for at least 11 of the 15 SNPs (N = 4209). In the Cox proportional hazards models non-cases were weighted by the inverse of their subcohort selection probabilities while cases were included with unit weights [16]. Time-to-event models were fitted using the survival package of R statistical software.

### Development of genetic risk scores

We used two approaches for deriving the genetic risk scores. The first derived a score for MORGAM participants using effect sizes from previous GWA studies (Genetic Risk Score 1; GRS1). A ‘weighted’ risk score was calculated for each subject by adding the number of risk alleles by SNP, multiplied by the associated effect size (log odds ratio) previously reported in the literature. Chosen SNPs were not in linkage disequilibrium (Table S2). The score assumed an additive risk model and no interaction between the SNPs. GRS1, comprised 11 SNPs and two haplotypes (Table S3) along with the Framingham score for classic risk factors.

Our second approach derived the coefficients for the score directly from the MORGAM prospective dataset. Score development was based on 1736 cases and 3082 non-cases. All SNPs were added in a model incorporating the Framingham score (GRS2). We extended the analysis by applying lasso, a penalized regression technique that carries out variable selection and estimates the coefficients of selected variables [27]. This form of penalization for overfitting removes redundant predictors from the model. SNPs with non-zero coefficients comprised score 3 (GRS3) along with the Framingham score. Lasso models were estimated using the glmnet package of R. Ten-year risk estimates for incident CHD events were derived from the fitted Cox regression models using the survival package of R.

### Risk model assessment

We used the MORGAM dataset to derive and test GRS2 and GRS3. The predictive value of the data-derived scores was evaluated using 10-fold cross-validation. This avoids using the same individuals in developing and testing the model. The dataset was split randomly into 10 equal sized validation sets and the prediction model was fitted to each of the 10 datasets obtained by leaving out each of the validation sets in turn, with the 10-year risk estimates for the omitted group derived from the model fitted to the remaining data [28]. While cross-validation is not a complete substitute for external validation, it utilises available data more efficiently than split sample validation [28] (Supporting Information S1). Validation of the data derived scores are based on individuals which have 10 years of follow up information (first 10 years from cohorts) comprising 632 cases and 1361 non-cases. To test whether the three genetic risk scores (GRS1–3) improve prediction, a baseline model with the Framingham score was compared to a model with each genetic risk score incorporated in the baseline model. Models were tested using the c-index improvement, integrated discrimination improvement (IDI) and net reclassification improvement (NRI) according to recently suggested risk limits 0% to <5%, 5% to <10%, 10% to <20% and ≥20% for 10 year risk limits [29]. In addition, ‘Clinical’ NRI quantifies the improvement in prediction in the intermediate risk group (5–20%) which incorporates a correction for the expected value of improvement [30]. This test measures the reclassification where only individuals in the intermediate group are tested with the GRS and have their risk recalculated. For exploring the effect of adding family history of CHD on the models, we studied the cohort definitions of family history (Table S4) and a separate coefficient for the family history covariate was estimated for each cohort. This was added to the Framingham model with genetic risk scores subsequently added to this model.

### GRS and event free survival analysis

Kaplan-Meier curves were plotted to depict CHD free survival by quarters of the genetic risk scores, and the log rank test was used to assess the differences. The log rank test estimated differences in survival curves across quarters of the externally derived GRS1 until age 70 and 10 years survival. While it was appropriate to calculate the significance of GRS1 as the risk sets were determined using externally derived coefficients we did not calculate it for the cross-validated scores.

### Population attributable fraction

Population attributable fraction (PAF) estimates the genetic risk scores' contribution in explaining CHD incidence in the MORGAM population. PAFs were calculated with respect to a hypothetical population genetic profile truncated from above the GRS population average. We defined population attributable risk as the population risk minus the modified risk when the GRS was set to the population mean, for those with above average GRS. Population attributable fraction is the ratio of this difference to the overall population risk, indicating the proportion of the risk ‘attributable’ to genetic variation. For reference, we also calculated PAF for continuous classic risk factor variables.

## Results

### Individual SNP/haplotype analysis

Cox regression analysis revealed four significant associations for additive allele effects with incident CHD in a model adjusting for cohort and Framingham score: rs1333049 (9p21); rs1256453 (PHACTR1); rs2048327 (LPA) and rs3008621 (MIA3) (Table 2). Unadjusted associations are given in Tables S5 and S6. Because rs9818870 (MRAS) was not significantly associated with CHD and not typed in the Swedish cohort, we excluded it from our GRS models. Sensitivity analysis excluding the Swedish cohort showed that the addition of this SNP did not appreciably change the results (see Supporting Information S1, Table S7).

### Genetic risk scores

#### Genetic risk score 1

The baseline model based on the Framingham Score achieved a c-index of 0.743. Adding GRS1 to the baseline model did not significantly improve discrimination (c-index 0.752, *p* = 0.11). However, risk classification improved significantly (NRI by 7.5% *p* = 0.017 and IDI by 0.4% *p* = 0.007) in the entire sample of men (Table 3, Table S8 for full reclassification statistics). Clinical NRI for men in the intermediate risk group only (431 cases, 664 non-cases) was not significant (6%, *p* = 0.17).

**Table 3:** Comparison of models with and without genetic risk scores.

	NRI			IDI		Clinical NRI	
	Value	SE	*p*	Value	*p*	Value	*p*
FRS+GRS1							
Cases	0.049	0.028	0.074	0.004	0.020	0.034	0.291
Non-cases	0.025	0.021	0.230	0.001	0.398	−0.029	0.435
	0.075	0.031	0.017	0.004	0.007	0.063	0.175
FRS+GRS2							
Cases	0.071	0.025	0.005	0.007	0.0003	0.061	0.042
Non-cases	0.007	0.021	0.755	0.0001	0.903	0.01	0.769
	0.065	0.032	0.044	0.007	0.0004	0.051	0.269
FRS+GRS3							
Cases	0.062	0.023	0.008	0.005	0.001	0.056	0.037
Non-cases	0.002	0.018	0.931	0.0002	0.721	−0.001	0.977
	0.060	0.029	0.039	0.005	0.0009	0.056	0.16

NRI measures reclassification across risk groups 0% to <5%, 5% to <10%, 10% to <20% and ≥20%, Clinical NRI measures the improvement for those in the middle (5 to <20%) risk group who are reclassified after inclusion of GRS. NRI Net Reclassification Index, IDI Integrated Discrimination Improvement, FRS+GRS Framingham risk score+genetic risk score.

#### Genetic risk scores 2 and 3

Score development (GRS2 and GRS3) was based on 1736 cases and 3082 non-cases. The validation set comprised 632 cases and 1361 non-cases from the first 10 years' of follow-up of the cohorts with a follow-up period of at least 10 years. For example, this was related to approximately 715 cases and 9575 non-cases in the full cohort using the case-cohort weighting incorporating censoring.

GRS2 comprised 15 SNPs with coefficients derived from a Cox model fitted to the MORGAM dataset (Table S3). While the LPA haplotypes were significantly associated with CHD in case-control studies they were not significant in our prospective dataset (Table 4) (or in a recent study evaluating CHD risk in diabetics [31]) and did not improve the data derived scores (data not shown). Instead, we utilised individual SNP data only at the LPA locus. GRS2 based on internally determined weight estimates gave broadly similar findings to GRS1. The c-index improved by 1.11% which was marginally significant (*p* = 0.048) when added to the baseline model. The overall NRI was 6.5% (*p* = 0.044) and IDI was 0.7% (*p* = 0.0004), almost all of this due to upward reclassification of cases (Table 3, Table S8). For the 431 cases and 664 non-cases initially in the intermediate risk category, clinical NRI was not significant (5.1% *p* = 0.2) but followed the trend in the whole group with cases correctly reclassified upwards (6.1%, *p* = 0.048). This indicates that while reclassification is mainly beneficial in the low and high risk groups, intermediate cases are correctly reclassified upwards to the high risk group.

**Table 4:** Association between LPA haplotypes and CHD in MORGAM.

rs2048327	rs3127599	rs7767084	rs10755578	Frequency	Haplotype combination in ref 21	HR reported in ref	CHD (1736 cases, 3082 non cases)	
							Pooled HR (95% C.I.)	*p* value
A	A	T	G	0.13	TTTG		0.99 (0.85–1.16)	0.94
G	G	T	C	0.03	CCTC	1.8	1.19 (0.88–1.61)	0.26
G	G	C	G	0.14	CCCG		1.12 (0.97–1.31)	0.12
A	A	T	C	0.03	TTTC		0.91 (0.65–1.27)	0.56
A	G	T	G	0.14	CTTG	1.2	1.14 (0.98–1.33)	0.09
G	G	T	G	0.02	CCTG		1.04 (0.74–1.47)	0.81

While MORGAM associations conditioned on a different allele set, haplotypic combinations are consistent with those reported in ref which are given here for comparison. Association was tested with a model adjusted for cohort and Framingham coefficients. Haplotypic ORs were used as coefficients for GRS1.

Lasso regression identified 8 variants with non-zero coefficients incorporated into genetic risk score 3 (Table S3). GRS3 demonstrated an improvement in the c-index from 0.742 to 0.755 (*p* = 0.0044), with a gain in NRI of 6% (*p* = 0.039) and an IDI of 0.5% (*p* = 0.0009 Table 3, Table S8). The clinical NRI for the intermediate risk men was not significant 5.6%, *p* = 0.16) but with cases correctly reclassified upward 5.5% *p* = 0.03). The c-index improvement of 1.22% resulting from addition of GRS3, compared to 1.11% from GRS2, indicates that only a small subset of 8 risk alleles are discriminatory in our models.

### Subgroup analysis in middle-aged men

We validated the prediction models in men aged 50–59 at baseline (436 cases and 603 non-cases). This controls the dominating effect of age on risk and indicates the potential benefit attainable in this group. Here, the Framingham risk score produced a c-index value of 0.661. C-index improvement was 2.6%, (*p* = 0.007) for GRS1, 2.8%, (*p* = 0.0038) for GRS2 and 2.6%, (*p* = 0.001) for GRS3. Corresponding NRI were 13.8% (*p* = 0.0022) for GRS1, 12.5%, (*p* = 0.0069) for GRS2 and 10.7%, (*p* = 0.015) for GRS3 with benefit mainly in cases reclassified upwards (Table S9).

### Addition of family history data to genetic risk scores

Adding family history to the baseline model for the whole group (627 cases, 1342 non-cases excluding missing information) improved NRI (5.5%, *p* = 0.023) but not the c-index (Table S10). Adding GRS1 or GRS2 to this model did not improve discrimination statistics. The estimated effect sizes for the family history covariate differed between the cohorts, being lower for FINRISK, a difference which cannot be explained by disparate definitions of family history. We thus focused on 50–59 year old men, since family history should become apparent by this stage and exclude parents of younger participants which have spent less time at risk of premature MI. The addition of family history information to the subgroup of men aged 50–59 significantly improved NRI (8.8%, *p* = 0.016) (Table 5). However it did not significantly improve the c-index (1.7%, *p* = 0.06) reflecting the relative insensitivity of this measure. Adding the genetic scores to a baseline Framingham model and family history continued to improve reclassification. Net reclassification was 9.8% (*p* = 0.019) for GRS1 and 13.2% (*p* = 0.015) for GRS2 with significant upward reclassification of cases (Table 5) indicating predictive improvement of early onset CHD events.

**Table 5:** Addition of family history information to genetic risk scores.

	NRI			IDI	
	Value	SE	*p*	Value	*p*
FRS+FH					
Cases	0.031	0.025	0.169	0.008	0.0005
Non-cases	0.053	0.026	0.044	0.001	0.469
	0.088	0.036	0.016	0.006	0.018
FRS+FH+GRS1					
Cases	0.065	0.035	0.065	0.006	0.0065
Non-cases	0.033	0.031	0.033	0.001	0.588
	0.098	0.042	0.019	0.007	0.003
FRS+FH+GRS2					
Cases	0.142	0.041	0.0005	0.018	0.001
Non-cases	0.011	0.039	0.787	0.001	0.683
	0.132	0.054	0.015	0.018	0.0025

Reclassification results comparing a baseline model including family history (FH); to models including genetic risk scores for men aged 50–59.

### Genetic risk scores and event free survival analysis

**Figure 2:**
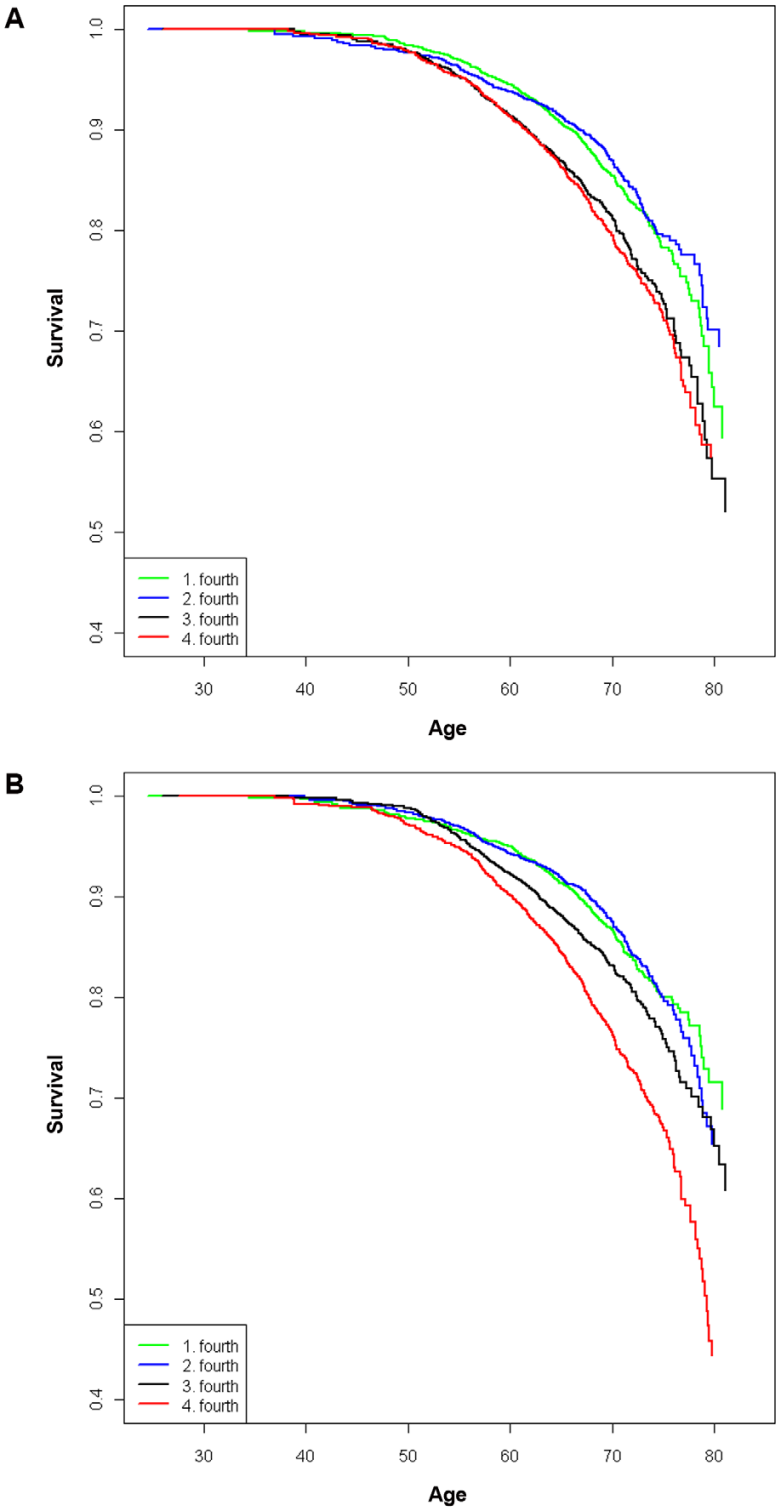
Kaplan-Meier curves may better predict those with premature CHD. Survival curves assessing the time to incident CHD with increasing age across four GRS categories for GRS1 (A) and GRS2 (B). Survival probabilities are truncated from 0–0.4.

Event free survival curves illustrate differences in risk of incident CHD across the quarters for GRS1 and GRS2 (Figure 2 A and B). The chance of reaching age 70 years event free was 80% in the quarter with the highest GRS1 and 86% in those with the lowest GRS1 (log rank test *p* = 0.001 for the difference). The chance of surviving ten years event free from the study baseline was 91% in the highest GRS1 group and 94% in the lowest GRS1 group (*p* = 0.001). Based on Figure 2, while GRS1 distinguishes the first and second quarter from the third and fourth groups, GRS2 clearly distinguishes the fourth or highest risk group from the other groups in terms of elevated risk. We also estimated event free 10-year survival in the 50–59 year old subgroup, as well as in the intermediate (5–20%) risk category based on the Framingham score. In the 50–59 year olds the 10-year survival probability in the highest (lowest) GRS1 quarter was 90% (94%, *p* = 0.003), and in the intermediate risk category 88% (91%, *p* = 0.01).

### Population attributable fraction

PAFs calculated the observed model-based risk compared to a hypothetical situation where men with above average genetic risk were moved to the population average genetic risk. The distribution of risk scores across the MORGAM cohorts were similar (Figure S2). The PAF for GRS1 was 12.1%, which serves only as a benchmark since it represents the external coefficients and not effects observed in the MORGAM data. However, PAFs for GRS2 and GRS3 were comparable to this: 11.9% (95% C.I. 8.8–15.4%) and 9.5% (5.1–13.6%) respectively. These PAF estimates are similar to the PAFs observed in MORGAM cohorts for HDL cholesterol (12.3% (95% C.I. 9.3–15.7%) and systolic BP (12.1% (9.5%–15.4%) while the PAF for diastolic BP and BMI were lower, (4.9% and 5.9%). (Table S11).

## Discussion

Recent success of genome wide association studies in identifying variants affecting disease risk stirred much debate about the utility of genetic risk scores for prediction of complex diseases. Scepticism was fuelled by results of often under-powered studies. Here, we demonstrate that genetic risk scores can meaningfully refine risk classification for coronary disease in men, particularly aged 50–59 years, when added to the information derived from conventional cardiovascular risk factors.

Other prospective cohorts evaluating the addition of genetic risk scores to classic risk factors have found only marginal benefits. A 13 SNP weighted score failed to significantly improve the c-index or NRI but showed marginal benefit for IDI (0.004, *p* = 0.0006) in Finnish and Swedish cohorts [8]. A weighted SNP score based on 29 CHD loci (although not directly comparable included the 13 loci used in our study) provided marginal reclassification benefit (NRI 2.8%, *p* = 0.03) in a prospective Dutch cohort but this benefit was mainly contributed by three SNPs [14]. A 13 SNP score failed to significantly improve the c-index or category led NRI but showed marginal reclassification benefit for continuous NRI (19% 95% C.I. 0.02–0.34) in an American cohort with European ancestry [13]. The addition of the newly discovered 16 SNPs failed to add significantly to prediction [13]. Our findings in the MORGAM prospectively followed European cohorts offer stronger effect estimates than observed in these recent studies [8], [13]–[14] which may need explanation. Firstly, we focused on men since the statistical power for showing effects in addition to classical risk factors may be enhanced when gender is excluded as a confounding factor. Moreover, our study included about 50% more incident coronary cases from across Europe [8], [13]–[14] Finally, we employed relative effect estimates from the largest GWA study performed to date for CHD [11], which were 1–6 percentage points smaller and thus resulted in more conservative estimates than those used by previous studies [8], [13].

### Genetic risk scores modestly improve prediction and particularly for middle-aged men

Applying the genetic risk score to all men reclassified between 6–7.5% of our cohort and in the subgroup of men aged 50–59 years reclassified between 10.7–13.8% with benefit mainly observed in identifying future cases. Age typically has a large impact on discrimination statistics, so when we limit the age range to 50–59 we limit the effect of age as the main CHD risk factor on the prediction models and the predictions become more sensitive to the effect of other factors such as the SNPs. The difference in the c-index between all men (0.743) and 50–59 year old men (0.661) is not widely recognised in clinical decision making but highlights the impact of the scores in this subgroup. From a clinical perspective, refinement in this group can aid decisions to initiate preventive measures and especially lifelong blood pressure or lipid lowering (statin) drug treatment which can be most challenging from an individual perspective and societal affordability.

Family history is an easily ascertainable risk factor, albeit sometimes uncertain [1]. A key requirement for any genetic risk score is that it should add predictive value over and above a family history [9]. As the reliability of family history may vary by age-group we focused this analysis on middle-aged men aged 50–59 where any family predisposition should have become apparent. In this subset, we found that the genetic risk scores improved reclassification even incorporating family history indicating that the two provide complementary and additive information on risk prediction.

While the effect of each risk allele is relatively modest, their importance to CHD development is significant, since their prevalence ranges from 10% to 87% in Europeans (Table 2). Survival curves illustrate that 25% of European men who carry the most risk alleles have a 9% risk of CHD over a 10 year period as compared to only 6% of those in the lowest quartile. From another perspective, a 20% risk of CHD is reached by age 67 years in those with the highest genetic risk compared to age 74 years by those with the lowest genetic risk score. The population attributable fraction for genetic risk as estimated by the score ranged from 9.8–12.7% which is similar to other risk factors such as HDL cholesterol, and systolic BP and highlights that currently identifiable genetic risk makes an important contribution to overall CHD risk.

### Comparison of the performance of genetic risk scores and biomarker risk scores

Novel blood biomarkers can measure subclinical features of cardiovascular disease capturing genetic and non-genetic components of disease and may provide an alternative to large scale population risk stratification. The MORGAM project found that the addition of a biomarker score comprising three novel biomarkers (NtProBNP, CRP, sensitive Troponin I) to a classic risk factors model improved 10-year risk estimation for cardiovascular events in middle aged European populations [32]. The biomarker risk model as well as the baseline risk model comprising classic risk factors were derived from FINRISK but externally validated in a subgroup of men aged 50–59 years in PRIME Belfast giving a c-index value of 0.67 for the baseline risk model. This value is similar to the baseline risk model based on the Framingham score for the 50–59 year old men in the present study which resulted in a c-index of 0.661. The similarity of the validation population in this biomarker study and the 50–59 year old validation subgroup allows drawing comparison between the two sets of results. The biomarker model resulted in a c-index improvement of 3% (*p* = 0.004) with NRI of 11% (*p* = 0.0008) which is very similar to the 2.6–2.8% improvement in the c-index and 10–13.7% improvement in NRI for the genetic risk scores. Because the genotypes of genetic risk scores are invariant, those with higher GRS may predispose individuals to disease earlier resulting in gradual increases in biomarker levels compared to those with lower GRS. Genetic risk scores could facilitate risk assessment earlier in life than is possible with phenotype-based tests when knowledge of classic risk factors is limited. However, given the heterogeneity in the behavioural responses to genetic risk perception [33], a full decision analysis would be needed to assess the cost effectiveness of screening middle aged men at the population level.

### Comparison of the performance of genetic risk scores as a predictor of CHD

We studied three genetic risk scores: GRS1 used effect estimates from previous GWA case-control studies, and two data-derived scores, GRS2 and GRS3, based on effect estimates from our prospectively collected data. Effect-sizes from GWA studies, such as that used in GRS1, may overestimate the relative risk derived from a combination of SNPs, since they often concern SNPs which are “winners” from a large discovery selection. Furthermore, most GWA studies are based on prevalent cases and the strength of the observed (and real) association of variants may differ between incident and prevalent disease. GRS2 was developed to allow for these potential confounders. GRS3 determined whether a more parsimonious sets of variants provided equivalent discrimination to the larger SNP set. All three scores improved 10 year CHD prediction in men in terms of reclassification, beyond that possible with baseline classic risk factors, while the data derived scores also improved the c-index significantly. While both approaches displayed differences in predicting risk, the overall performance was similar suggesting that adding predictive SNPs to prognostic models may be beneficial even if the effect estimates are not perfectly accurate. The lasso method, resulted in a more parsimonious score (GRS3) which had comparable predictive power to GRS2, indicating that model improvement was mostly due to 8 SNPs.

### Study strengths and limitations

Despite studying a large representation of the European population, further validation is required in larger populations with different levels of absolute risk and other ethnic groups. This applies specifically to GRS2 and GRS3, in which effect estimates were derived in the same population. Moreover, the list of genetic variants associated with CHD is likely to increase such that our scores do not capture the full potential of incorporating genetic information. Thus, our findings can be only a starting point for future analyses with other cohorts to refine the predictive value of such genetic scores.

### Conclusions

Our findings demonstrate that adding a genetic risk score to classic risk factors may improve CHD prediction. Moreover, future attempts to add precision to a score may be of greater benefit to population subgroups [31], here especially in middle-aged men. As the costs of obtaining genetic information fall, incorporating such information could make an important contribution in more accurately directing primary prevention measures and reducing the burden of CHD.

## Supporting Information

Table S1 Background characteristics of the nine cohorts given for cases and non-cases. Data are mean (SD) or number %. NA = not available. As ATBC and MONICA-KORA did not collect information on current drug therapy, these were considered as ‘no medication’ for the analysis.
(DOCX)

Table S2 Average correlations between SNPs are calculated across the different centres weighted by the subcohort size (N = 3390).
(DOCX)

Table S3 β coefficients for each genetic risk score. HAGTG and HGGTC refer to LPA haplotypes.
(DOCX)

Table S4 Definitions of self reported family history of CHD/MI used in the MORGAM cohorts.
(DOCX)

Table S5 Association between SNPs and coronary heart disease. Univariate associations of the SNPs were tested with Cox proportional hazards model adjusted for sex, area/cohort. We investigated the model fit with alternative models of effect (dominant, additive, recessive) operating at each locus.
(DOCX)

Table S6 Haplotypes reported by ref 20 covering the gene region SLC22A3, LPAL2, LPA and their association with CHD in MORGAM. While the association analysis conditioned on a different set of alleles in MORGAM, these combinations are consistent with those reported in the ref which are given here for comparison. The association was tested with Cox proportional hazards model adjusted for area and sex. This model estimates their effect sizes from simultaneously including all haplotypes compared to the most frequent TCTC haplotype.
(DOCX)

Table S7 Net reclassification results for the comparison of a baseline model including Framingham coefficients and area to a model including genetic risk scores (GRS1 including the MRAS SNP and the baseline model. Genetic risk scores have been derived in all men and men aged 50–59 years at baseline.
(DOCX)

Table S8 Net reclassification tables for comparison of a baseline model including Framingham coefficients and area to a model including genetic risk scores (GRS1, GRS2 and GRS3 respectively) and the baseline model. Numbers are estimated from a model originally based on 10 year predicted risk on the basis of FRS score, accounting for case-cohort weighting and censoring, e.g. GRS1 was related to approximately 715 cases and 9575 non-cases in the full cohort, see corresponding summary details in Table 3.
(DOCX)

Table S9 Net reclassification results for the comparison of a baseline model including Framingham coefficients and area to a model including genetic risk scores (GRS1, GRS2 and GRS3 respectively) and the baseline model. Genetic risk scores have been derived in all men and validated in men aged 50–59 at baseline).
(DOCX)

Table S10 Reclassification results comparing a baseline model including family history (FH); to models including genetic risk scores for all men (627 cases, 1342 non-cases).
(DOCX)

Table S11 Ten year risk of CHD in the population is calculated for a baseline model adjusted for area for 1736 cases and 3082 non-cases related back to the full cohort. Ten year risk with modified risk of each risk factor is calculated by bringing those with above average risk factor values to the population mean and calculating the difference to estimate the proportion of risk attributable to that risk factor. Standard errors and confidence intervals for the statistics were calculated from 2500 bootstrap replications.
(DOCX)

Supporting Information S1 (DOCX)
