# Supplementary material for: Genetic Markers Enhance Coronary Risk Prediction in Men: The MORGAM Prospective Cohorts
Source: PLoS One. 2012 Jul 25;7(7):e40922. doi: 10.1371/journal.pone.0040922 (PMC3405046; doi:10.1371/journal.pone.0040922)
Supplement: Table S1 — Background characteristics of the nine cohorts given for cases and non-cases. Data are mean (SD) or number %. NA = not available. As ATBC and MONICA-KORA did not collect information on current drug therapy, these were considered as ‘no medication’ for the analysis. (DOCX) [file pone.0040922.s001.docx]

|  | FINRISK Eastern Finland | | FINRISK South western Finland | | ATBC, Southern Finland | | PRIME Lille, France | | PRIME Toulouse France | |
| --- | --- | --- | --- | --- | --- | --- | --- | --- | --- | --- |
| *Baseline characteristics* | *cases* | *non-cases* | *cases* | *non-cases* | *cases* | *non-cases* | *cases* | *non-cases* | *cases* | *non-cases* |
| Total number | 254 | 3137 | 184 | 2633 | 436 | 4129 | 137 | 2221 | 145 | 2268 |
| Age (years) | 57.0 (9.3) | 45.6 (12.3) | 57.7 (9.3) | 46.6 (12.9) | 64.1 (4.9) | 62.6 (4.9) | 55.8 (2.9) | 55.1 (2.9) | 55.1 (2.9) | 54.9 (2.8) |
| Cholesterol |  |  |  |  |  |  |  |  |  |  |
| Total, mmol/L | 6.11 (1.04) | 5.60 (1.07) | 5.97 (1.15) | 5.50 (1.06) | 6.09 (1.05) | 5.92 (1.03) | 6.06 (1.00) | 5.73 (0.95) | 5.83 (0.92) | 5.50 (0.89) |
| HDL mmol/L | 1.19 (0.30) | 1.27 (0.32) | 1.16 (0.30) | 1.26 (0.31) | 1.11 (0.30) | 1.18 (0.31) | 1.28 (0.36) | 1.34 (0.33) | 1.14 (0.30) | 1.25 (0.30) |
| Ratio of total to HDL | 5.42 (1.49) | 4.65 (1.50) | 5.47 (1.77) | 4.62 (1.45) | 5.96 (3.18) | 5.34 (1.69) | 5.02 (1.42) | 4.54 (1.50) | 5.49 (1.71) | 4.63 (1.38) |
| Blood pressure |  |  |  |  |  |  |  |  |  |  |
| Systolic (mm Hg) | 150.6 (21.2) | 138.7 (18.3) | 146.4 (18.6) | 137.7 (17.8) | 145.0 (19.4) | 141.1 (18.2) | 145.9 (19.2) | 138.3 (19.0) | 130.1 (15.8) | 126.4 (14.7) |
| Diastolic (mm Hg) | 86.7 (11.2) | 83.6 (11.8) | 88.3 (10.6) | 84.7 (11.7) | 84.9 (9.5) | 84.9 (10.2) | 90.1 (12.6) | 86.3 (12.1) | 82.0 (10.3) | 79.3 (9.7) |
| BMI (kg/m^2^) | 28.4 (3.9) | 26.9 (4.0) | 28.2 (3.5) | 26.6 (3.8) | 27.0 (4.2) | 26.8 (4.1) | 27.6 (3.9) | 26.5 (3.5) | 26.8 (3.6) | 26.2 (3.1) |
| Daily smoker | 83 (32.7) | 896 (28.6) | 59 (32.1) | 790 (30.0) | 343 (78.7) | 3226 (78.1) | 30 (21.9) | 399 (18.0) | 40 (27.6) | 403 (17.8) |
| Current drug therapy |  |  |  |  |  |  |  |  |  |  |
| Lipid lowering | 12 (4.7) | 71 (2.3) | 12 (6.5) | 56 (2.1) | NA | NA | 25 (18.2) | 285 (12.8) | 23 (15.9) | 307 (13.5) |
| High BP medication | 70 (27.6) | 354 (11.3) | 42 (22.8) | 251 (9.5) | NA | NA | 33 (24.1) | 332 (14.9) | 35 (24.1) | 293 (12.9) |
| History of diabetes | 30 (11.8) | 138 (4.4) | 27 (14.7) | 82 (3.1) | 39 (8.9) | 194 (4.7) | 12 (8.8) | 115 (5.2) | 15 (10.3) | 91 (4.0) |
| History of hypertension | 126 (49.6) | 1062 (33.9) | 83 (45.1) | 814 (30.9) | NA | NA | 44 (32.1) | 542 (24.4) | 47 (32.4) | 549 (24.2) |
| Prevalent cases N, % |  |  |  |  |  |  |  |  |  |  |
| stroke | 13 (5.1) | 62 (2.0) | 10 (5.4) | 48 (1.8) | 43 (9.9) | 151 (3.7) | 2 (1.5) | 16 (0.7) | 1 (0.7) | 8 (0.4) |
| Family history of CHD | 86 (33.9) | 838 (26.7) | 36 (19.6) | 495 (18.8) | NA | NA | 22 (16.1) | 183 (8.2) | 16 (11.0) | 160 (7.1) |
| *Incident CHD events during follow-up N, %* | | |  |  |  |  |  |  |  |  |
| men | 254 | 0 | 184 | 0 | 436 | 0 | 137 | 0 | 145 | 0 |
| *Random subsample of the case cohort set* | | |  |  |  |  |  |  |  |  |
| men | 54 (21.3) | 350 (11.2) | 32 (17.4) | 266 (10.1) | 92 (21.1) | 869 (21.0) | 3 (2.2) | 113 (5.1) | 7 (4.8) | 114 (5.0) |

Table S1. Background characteristics of the nine cohorts given for cases and non-cases.  Data are mean (SD) or number %. NA=not available. As ATBC and MONICA-KORA did not collect information on current drug therapy, these were considered as ‘no medication’ for the analysis.

|  | PRIME Strasbourg France | | PRIME Belfast UK | | MONICA KORA Augsburg Germany | | Northern Sweden | |
| --- | --- | --- | --- | --- | --- | --- | --- | --- |
| *Baseline characteristics* | *cases* | *non-cases* | *cases* | *non-cases* | *cases* | *non-cases* | *cases* | *non-cases* |
| Total number | 131 | 2145 | 106 | 2437 | 304 | 4201 | 39 | 1314 |
| Age (years) | 55.2 (3.1) | 54.7 (2.9) | 55.3 (2.8) | 54.7 (2.9) | 57.7 (8.3) | 53.0 (10.9) | 52.3 (7.2) | 45.0 (11.2) |
| Cholesterol |  |  |  |  |  |  |  |  |
| Total, mmol/L | 6.14 (1.02) | 5.78 (0.99) | 6.11 (1.11) | 5.87 (1.02) | 6.58 (1.16) | 6.18 (1.18) | 6.70 (1.05) | 6.21 (1.25) |
| HDL mmol/L | 1.16 (0.30) | 1.27 (0.34) | 1.11 (0.27) | 1.19 (0.32) | 1.22 (0.37) | 1.30 (0.39) | 1.22 (0.25) | 1.26 (0.32) |
| Ratio of total to HDL | 5.60 (1.71) | 4.87 (1.59) | 5.84 (1.84) | 5.24 (1.56) | 5.83 (1.99) | 5.30 (5.31) | 5.70 (1.45) | 5.22 (1.65) |
| Blood pressure |  |  |  |  |  |  |  |  |
| Systolic (mm Hg) | 142.6 (19.5) | 134.6 (18.2) | 141.3 (23.6) | 133.7 (20.2) | 143.7 (21.0) | 138.0 (18.4) | 137.2 (18.9) | 129.3 (16.8) |
| Diastolic (mm Hg) | 89.9 (12.2) | 86.5 (11.0) | 85.7 (10.9) | 81.7 (11.5) | 84.2 (12.4) | 83.9 (11.4) | 88.3 (14.2) | 82.4 (11.1) |
| BMI (kg/m^2^) | 28.1 (3.4) | 27.3 (3.5) | 26.8 (3.2) | 26.2 (3.4) | 28.0 (3.8) | 27.4 (3.5) | 27.5 (2.9) | 25.9 (3.5) |
| Daily smoker | 31 (23.7) | 352 (16.4) | 37 (34.9) | 554 (22.7) | 127 (41.8) | 1079 (25.7) | 19 (48.7) | 258 (19.6) |
| Current drug therapy |  |  |  |  |  |  |  |  |
| Lipid lowering | 19 (14.5) | 209 (9.7) | 0 (0.0) | 30 (1.2) | NA | NA | 1 (2.6) | 7 (0.5) |
| High BP medication | 29 (22.1) | 254 (11.8) | 10 (9.4) | 213 (8.7) | 69 (22.7) | 455 (10.8) | 6 (15.4) | 72 (5.5) |
| History of diabetes | 8 (6.1) | 92 (4.3) | 5 (4.7) | 43 (1.8) | 46 (15.1) | 191 (4.5) | 3 (7.7) | 27 (2.1) |
| History of hypertension | 44 (33.6) | 461 (21.5) | 19 (17.9) | 378 (15.5) | 131 (43.1) | 1244 (29.6) | 16 (41.0) | 208 (15.8) |
| Prevalent cases N, % |  |  |  |  |  |  |  |  |
| stroke | 1 (0.8) | 14 (0.7) | 1 (0.9) | 14 (0.6) | 14 (4.6) | 64 (1.5) | 3 (7.7) | 14 (1.1) |
| Family history of CHD | 18 (13.7) | 118 (5.5) | 35 (33.0) | 414 (17.0) | 71 (23.4) | 730 (17.4) | 7 (17.9) | 125 (9.5) |
| *Incident CHD events during follow-up N, %* | | |  |  |  |  |  |  |
| men | 131 | 0 | 106 | 0 | 304 | 0 | 39 | 0 |
| *Random subsample of the case cohort set* | | |  |  |  |  |  |  |
| men | 13 (9.9) | 112 (5.2) | 9 (8.5) | 151 (6.2) | 95 (31.2) | 1026 (24.4) | 3 (7.7) | 81 (6.2) |

Table S1 continued. Background characteristics of the nine cohorts given for cases and non-cases.  Data are mean (SD) or number %. NA=not available. As ATBC and MONICA-KORA did not collect information on current drug therapy, these were considered as ‘no medication’ for the analysis.

Supplementary Table 2 Average correlations between SNPs are calculated across the different centres weighted by the subcohort size (N=3596).
