# Supplementary material for: Genetic Markers Enhance Coronary Risk Prediction in Men: The MORGAM Prospective Cohorts
Source: PLoS One. 2012 Jul 25;7(7):e40922. doi: 10.1371/journal.pone.0040922 (PMC3405046; doi:10.1371/journal.pone.0040922)
Supplement: Table S2 — Average correlations between SNPs are calculated across the different centres weighted by the subcohort size (N = 3390). (DOCX) [file pone.0040922.s002.docx]

| **SNP** | **rs10755578** | **rs11206510** | **rs1122608** | **rs12526453** | **rs1333049** | **rs2048327** | **rs2259816** | **rs3008621** | **rs3127599** | **rs3184504** | **rs501120** | **rs646776** | **rs6725887** | **rs7767084** | **rs9818870** | **rs9982601** |
| --- | --- | --- | --- | --- | --- | --- | --- | --- | --- | --- | --- | --- | --- | --- | --- | --- |
| **rs10755578** | 1 | 0.05 | 0.03 | 0.03 | 0.04 | 0.60 | 0.03 | 0.04 | 0.57 | 0.06 | 0.04 | 0.03 | 0.03 | 0.45 | 0.02 | 0.05 |
| **rs11206510** | 0.05 | 1 | 0.03 | 0.04 | 0.03 | 0.06 | 0.03 | 0.03 | 0.07 | 0.04 | 0.02 | 0.04 | 0.02 | 0.05 | 0.04 | 0.05 |
| **rs1122608** | 0.03 | 0.03 | 1 | 0.03 | 0.03 | 0.04 | 0.03 | 0.03 | 0.03 | 0.05 | 0.05 | 0.03 | 0.04 | 0.03 | 0.07 | 0.04 |
| **rs12526453** | 0.03 | 0.04 | 0.03 | 1 | 0.04 | 0.05 | 0.04 | 0.03 | 0.03 | 0.03 | 0.02 | 0.04 | 0.04 | 0.02 | 0.04 | 0.03 |
| **rs1333049** | 0.04 | 0.03 | 0.03 | 0.04 | 1 | 0.04 | 0.03 | 0.03 | 0.05 | 0.03 | 0.04 | 0.03 | 0.05 | 0.03 | 0.02 | 0.03 |
| **rs2048327** | 0.60 | 0.06 | 0.04 | 0.05 | 0.04 | 1 | 0.06 | 0.05 | 0.13 | 0.05 | 0.03 | 0.02 | 0.03 | 0.56 | 0.04 | 0.04 |
| **rs2259816** | 0.03 | 0.03 | 0.03 | 0.04 | 0.03 | 0.06 | 1 | 0.05 | 0.04 | 0.03 | 0.04 | 0.03 | 0.03 | 0.07 | 0.03 | 0.04 |
| **rs3008621** | 0.04 | 0.03 | 0.03 | 0.03 | 0.03 | 0.05 | 0.05 | 1 | 0.04 | 0.04 | 0.05 | 0.03 | 0.02 | 0.03 | 0.03 | 0.05 |
| **rs3127599** | 0.57 | 0.07 | 0.03 | 0.03 | 0.05 | 0.13 | 0.04 | 0.04 | 1 | 0.05 | 0.02 | 0.06 | 0.05 | 0.28 | 0.04 | 0.03 |
| **rs3184504** | 0.06 | 0.04 | 0.05 | 0.03 | 0.03 | 0.05 | 0.03 | 0.04 | 0.05 | 1 | 0.02 | 0.03 | 0.03 | 0.03 | 0.05 | 0.04 |
| **rs501120** | 0.04 | 0.02 | 0.05 | 0.02 | 0.04 | 0.03 | 0.04 | 0.05 | 0.02 | 0.02 | 1 | 0.03 | 0.05 | 0.03 | 0.04 | 0.03 |
| **rs646776** | 0.03 | 0.04 | 0.03 | 0.04 | 0.03 | 0.02 | 0.03 | 0.03 | 0.06 | 0.03 | 0.03 | 1 | 0.05 | 0.04 | 0.04 | 0.03 |
| **rs6725887** | 0.03 | 0.02 | 0.04 | 0.04 | 0.05 | 0.03 | 0.03 | 0.02 | 0.05 | 0.03 | 0.05 | 0.05 | 1 | 0.03 | 0.04 | 0.04 |
| **rs7767084** | 0.45 | 0.05 | 0.03 | 0.02 | 0.03 | 0.56 | 0.07 | 0.03 | 0.28 | 0.03 | 0.03 | 0.04 | 0.03 | 1 | 0.03 | 0.03 |
| **rs9818870** | 0.02 | 0.04 | 0.07 | 0.04 | 0.02 | 0.04 | 0.03 | 0.03 | 0.04 | 0.05 | 0.04 | 0.04 | 0.04 | 0.03 | 1 | 0.04 |
| **rs9982601** | 0.05 | 0.05 | 0.04 | 0.03 | 0.03 | 0.04 | 0.04 | 0.05 | 0.03 | 0.04 | 0.03 | 0.03 | 0.04 | 0.03 | 0.04 | 1 |

Table S2 Average correlations between SNPs are calculated across the different centres weighted by the subcohort size (N=3390).
