# Supplementary material for: Genetic Markers Enhance Coronary Risk Prediction in Men: The MORGAM Prospective Cohorts
Source: PLoS One. 2012 Jul 25;7(7):e40922. doi: 10.1371/journal.pone.0040922 (PMC3405046; doi:10.1371/journal.pone.0040922)
Supplement: Table S3 — β coefficients for each genetic risk score. HAGTG and HGGTC refer to LPA haplotypes. (DOCX) [file pone.0040922.s003.docx]

| SNP | GRS1 | GRS2 | GRS3 |
| --- | --- | --- | --- |
| rs3008621 | -0.095 | -0.163 | -0.098 |
| rs11206510 | -0.077 | 0.012 | 0 |
| rs646776 | 0.174 | 0.110 | 0.059 |
| rs6725887 | 0.131 | 0.030 | 0 |
| rs2048327 | NA | -0.125 | -0.095 |
| rs3127599 | NA | -0.033 | 0 |
| rs7767084 | NA | -0.007 | 0 |
| rs10755578 | NA | -0.036 | -0.0001 |
| HAGTG | 0.182 | NA | NA |
| HGGTC | 0.599 | NA | NA |
| rs12526453 | 0.095 | 0.138 | 0.095 |
| rs1333049 | 0.182 | 0.243 | 0.206 |
| rs501120 | -0.104 | 0.092 | 0.039 |
| rs3184504 | -0.067 | 0.023 | 0 |
| rs2259816 | 0.077 | 0.007 | 0 |
| rs1122608 | 0.131 | -0.012 | 0 |
| rs9982601 | -0.165 | -0.082 | -0.003 |

Table S3. β coefficients for each genetic risk score. HAGTG and HGGTC refer to LPA haplotypes.
