# Supplementary material for: Genetic Markers Enhance Coronary Risk Prediction in Men: The MORGAM Prospective Cohorts
Source: PLoS One. 2012 Jul 25;7(7):e40922. doi: 10.1371/journal.pone.0040922 (PMC3405046; doi:10.1371/journal.pone.0040922)
Supplement: Table S4 — Definitions of self reported family history of CHD/MI used in the MORGAM cohorts. (DOCX) [file pone.0040922.s004.docx]

| cohort | relationship with participant | premature myocardial infarction |
| --- | --- | --- |
| PRIME | parents and siblings | <55 men, <65 women |
| FINRISK92 | parents | <60 men and women |
| FINRISK97 | parents and siblings | <60 men and women |
| Augsburg | parents | no age limit |
| Sweden | parents, siblings, parents' siblings | <65 men and women |

Table S4. Definitions of self reported family history of CHD/MI used in the MORGAM cohorts.
