# Supplementary material for: Genetic Markers Enhance Coronary Risk Prediction in Men: The MORGAM Prospective Cohorts
Source: PLoS One. 2012 Jul 25;7(7):e40922. doi: 10.1371/journal.pone.0040922 (PMC3405046; doi:10.1371/journal.pone.0040922)
Supplement: Table S5 — Association between SNPs and coronary heart disease. Univariate associations of the SNPs were tested with Cox proportional hazards model adjusted for sex, area/cohort. We investigated the model fit with alternative models of effect (dominant, additive, recessive) operating at each locus. (DOCX) [file pone.0040922.s005.docx]

| SNP | CHD (1736 cases, 3082 non cases) | |
| --- | --- | --- |
|  | Pooled HR (95% C.I.) | *p* value |
| rs3008621 | 0.87 (0.75, 1.01) | 0.062 |
| rs11206510 | 0.98 (0.87, 1.11) | 0.770 |
| rs646776 | 1.11 (0.99, 1.24) | 0.070 |
| rs9818870 | 0.92 (0.79, 1.07) | 0.270 |
| rs6725887 | 0.99 (0.87, 1.14) | 0.980 |
| rs2048327 | 0.88 (0.810, 0.97) | 0.017 |
| rs3127599 | 1.02 (0.92, 1.12) | 0.690 |
| rs7767084 | 1.08 (0.95, 1.22) | 0.220 |
| rs10755578 | 0.92 (0.83, 1.01) | 0.073 |
| rs12526453 | 1.11 (0.99, 1.22) | 0.052 |
| rs1333049 | 1.29 (1.18, 1.42) | 9.7E-8 |
| rs501120 | 1.11 (0.95, 1.13) | 0.180 |
| rs3184504 | 1.03 (0.94, 1.13) | 0.540 |
| rs2259816 | 0.98 (0.90, 1.08) | 0.780 |
| rs1122608 | 1.01 (0.91, 1.13) | 0.890 |
| rs9982601 | 0.96 (0.84, 1.09) | 0.510 |

Table S5. Association between SNPs and coronary heart disease. Univariate associations of the SNPs were tested with Cox proportional hazards model adjusted for sex, area/cohort. We investigated the model fit with alternative models of effect (dominant, additive, recessive) operating at each locus.
