# Supplementary material for: Genetic Markers Enhance Coronary Risk Prediction in Men: The MORGAM Prospective Cohorts
Source: PLoS One. 2012 Jul 25;7(7):e40922. doi: 10.1371/journal.pone.0040922 (PMC3405046; doi:10.1371/journal.pone.0040922)
Supplement: Table S6 — Haplotypes reported by ref 20 covering the gene region SLC22A3, LPAL2, LPA and their association with CHD in MORGAM. While the association analysis conditioned on a different set of alleles in MORGAM, these combinations are consistent with those reported in the ref which are given here for comparison. The association was tested with Cox proportional hazards model adjusted for area and sex. This model estimates their effect sizes from simultaneously including all haplotypes compared to the most frequent TCTC haplotype. (DOCX) [file pone.0040922.s006.docx]

| rs2048327 | rs3127599 | rs7767084 | rs10755578 | Haplotype combination in ref 21 | p | HR (95% CI) |
| --- | --- | --- | --- | --- | --- | --- |
|  |  |  |  |  |  |  |
| A | A | T | G | TTTG | 0.81 | 1.02 (0.88, 1.17) |
| G | G | T | C | CCTC | 0.2 | 1.2 (0.91, 1.59) |
| G | G | C | G | CCCG | 0.14 | 1.11 (0.97, 1.28) |
| A | A | T | C | TTTC | 0.44 | 0.88 (0.65, 1.21) |
| A | G | T | G | CTTG | 0.05 | 1.15 (1.0, 1.32) |
| G | G | T | G | CCTG | 0.97 | 1.01 (0.72, 1.39) |

Table S6. Haplotypes reported by ref 21 covering the gene region SLC22A3, LPAL2, LPA and their association with CHD in MORGAM. While the association analysis conditioned on a different set of alleles in MORGAM, these combinations are consistent with those reported in the ref which are given here for comparison. The association was tested with Cox proportional hazards model adjusted for area and sex. This model estimates their effect sizes from simultaneously including all haplotypes compared to the most frequent TCTC haplotype.
