# Supplementary material for: Genetic Markers Enhance Coronary Risk Prediction in Men: The MORGAM Prospective Cohorts
Source: PLoS One. 2012 Jul 25;7(7):e40922. doi: 10.1371/journal.pone.0040922 (PMC3405046; doi:10.1371/journal.pone.0040922)
Supplement: Table S7 — Net reclassification results for the comparison of a baseline model including Framingham coefficients and area to a model including genetic risk scores (GRS1 including the MRAS SNP and the baseline model. Genetic risk scores have been derived in all men and men aged 50–59 years at baseline. (DOCX) [file pone.0040922.s007.docx]

|  |  | NRI | | | IDI | | Clinical NRI | |
| --- | --- | --- | --- | --- | --- | --- | --- | --- |
|  |  | Value | SE | *p* | Value | *p* | Value | *p* |
| all men | FRS + GRS1 |  |  |  |  |  |  |  |
|  | Cases | 0.039 | 0.028 | 0.155 | 0.003 | 0.090 | 0.026 | 0.413 |
|  | Non-cases | -0.037 | 0.021 | 0.087 | -0.001 | 0.297 | -0.041 | 0.289 |
|  |  | 0.076 | 0.032 | 0.017 | 0.004 | 0.032 | 0.067 | 0.159 |
| 50-59 year old men | FRS + GRS1 |  |  |  |  |  |  |  |
|  | Cases | 0.076 | 0.034 | 0.027 | 0.006 | 0.0081 |  |  |
|  | Non-cases | -0.054 | 0.036 | 0.135 | -0.0008 | 0.557 |  |  |
|  |  | 0.129 | 0.045 | 0.004 | 0.006 | 0.0033 |  |  |

Table S7 Reclassification results for the comparison of a baseline model including Framingham coefficients and area to a model including genetic risk scores (GRS1 including the MRAS SNP and the baseline model. Genetic risk scores have been derived in all men and men aged 50-59 years at baseline.
