# Supplementary material for: Genetic Markers Enhance Coronary Risk Prediction in Men: The MORGAM Prospective Cohorts
Source: PLoS One. 2012 Jul 25;7(7):e40922. doi: 10.1371/journal.pone.0040922 (PMC3405046; doi:10.1371/journal.pone.0040922)
Supplement: Table S8 — Net reclassification tables for comparison of a baseline model including Framingham coefficients and area to a model including genetic risk scores (GRS1, GRS2 and GRS3 respectively) and the baseline model. Numbers are estimated from a model originally based on 10 year predicted risk on the basis of FRS score, accounting for case-cohort weighting and censoring, e.g. GRS1 was related to approximately 715 cases and 9575 non-cases in the full cohort, see corresponding summary details in Table 3. (DOCX) [file pone.0040922.s008.docx]

|  | **Predicted** | **Genetic risk score 1** | | | | **Reclassified** | |
| --- | --- | --- | --- | --- | --- | --- | --- |
|  | **risk** | **0-5%** | **5-10%** | **10-20%** | **≥20%** | **up** | **down** |
| **Cases** | **0-5%** | 107 (72.3%) | 40 (27.0%) | 1 (0.7%) | 0 | 113 | 80 |
|  | **5-10%** | 25 (10.4%) | 175 (72.7%) | 40 (16.8%) | 0 | 16.5% | 11.5% |
|  | **10-20%** | 0 | 39 (17.5%) | 155 (68.8%) | 31 (13.8%) |  |  |
|  | **≥20%** | 0 | 0 | 15 (21.3%) | 56 (78.7%) |  |  |
|  | **0-5%** | 4864 (91.4%) | 445 (8.4%) | 12 (0.2%) | 0 | 883 | 1125 |
| **Non-** | **5-10%** | 714 (25.2%) | 1771 (62.6%) | 343 (12.1%) | 3 (0.1%) | 9.2% | 11.8% |
| **cases** | **10-20%** | 4 (0.3%) | 341 (29.0%) | 750 (63.8%) | 81 (6.9%) |  |  |
|  | **≥20%** | 0 | 0 | 65 (26.2%) | 183 (73.8%) |  |  |
|  |  |  |  |  |  |  |  |
|  | **Predicted** | **Genetic risk score 2** | | | | **Reclassified** | |
|  | **risk** | **0-5%** | **5-10%** | **10-20%** | **≥20%** | **up** | **down** |
| **Cases** | **0-5%** | 111 (74.6%) | 37 (25.3%) | 0 (0.1%) | 0 | 115 | 68 |
|  | **5-10%** | 21 (8.7%) | 176 (72.7%) | 45 (18.6%) | 0 | 17% | 9.9% |
|  | **10-20%** | 0 | 35 (15.3%) | 158 (70.2%) | 33 (14.5%) |  |  |
|  | **≥20%** | 0 | 0 | 13 (17.9%) | 57 (82.1%) |  |  |
|  | **0-5%** | 4662 (87.6%) | 657 (12.3%) | 2 (0.0%) | 0 | 1033 | 970 |
| **Non-** | **5-10%** | 612 (21.6%) | 1906 (67.4%) | 312 (11.0%) | 0 | 10.8% | 10.1% |
| **cases** | **10-20%** | 0 | 294 (25.0%) | 818 (69.6%) | 63 (5.4%) |  |  |
|  | **≥20%** | 0 | 0 | 63 (25.5%) | 185 (74.5%) |  |  |
|  |  |  |  |  |  |  |  |
|  | **Predicted** | **Genetic risk score 3** | | | | **Reclassified** | |
|  | **risk** | **0-5%** | **5-10%** | **10-20%** | **≥20%** | **up** | **down** |
| **Cases** | **0-5%** | 116 (78.4%) | 32 (21.6%) | 0 | 0 | 87 | 47 |
|  | **5-10%** | 14 (5.6%) | 193 (79.9%) | 35 (14.4%) | 0 | 13% | 6.8% |
|  | **10-20%** | 0 | 24 (10.6%) | 181 (80.4%) | 20 (9.0%) |  |  |
|  | **≥20%** | 0 | 0 | 9 (13.3%) | 61 (86.7%) |  |  |
|  | **0-5%** | 4813 (90.5%) | 507 (9.5%) | 0 | 0 | 781 | 765 |
| **Non-** | **5-10%** | 471 (16.6%) | 2141 (75.6%) | 219 (7.7%) | 0 | 8.1% | 8% |
| **cases** | **10-20%** | 0 | 244 (20.8%) | 877 (74.6%) | 55 (4.7%) |  |  |
|  | **≥20%** | 0 | 0 | 50 (20.1%) | 198 (79.9%) |  |  |

Table S8 Net reclassification tables for comparison of a baseline model including Framingham coefficients and area to a model including genetic risk scores (GRS1, GRS2 and GRS3 respectively) and the baseline model. NRI risk limits are 0% to <5%, 5% to <10%, 10% to <20% and ≥20%. Numbers are estimated from a model originally based on 10 year predicted risk on the basis of FRS score, accounting for case-cohort weighting and censoring, e.g. GRS1 was related to approximately 715 cases and 9575 non-cases in the full cohort, see corresponding summary details in Table 3.
