# Supplementary material for: Genetic Markers Enhance Coronary Risk Prediction in Men: The MORGAM Prospective Cohorts
Source: PLoS One. 2012 Jul 25;7(7):e40922. doi: 10.1371/journal.pone.0040922 (PMC3405046; doi:10.1371/journal.pone.0040922)
Supplement: Table S9 — Net reclassification results for the comparison of a baseline model including Framingham coefficients and area to a model including genetic risk scores (GRS1, GRS2 and GRS3 respectively) and the baseline model. Genetic risk scores have been derived in all men and validated in men aged 50–59 at baseline). (DOCX) [file pone.0040922.s009.docx]

| Supplementary Table 5. β coefficients for each genetic risk score. HAGTG and HGGTC refer to LPA haplotypes. | NRI | | IDI | | c-index | |
| --- | --- | --- | --- | --- | --- | --- |
|  | Value | *p* | Value | *p* | Difference | *p* |
| FRS + GRS1 |  |  |  |  |  |  |
| Cases | 0.094 | 0.0123 | 0.006 | 0.0017 |  |  |
| Non-cases | 0.048 | 0.194 | 0.001 | 0.578 |  |  |
|  | 0.138 | 0.0022 | 0.007 | 0.0006 | 2.6% | 0.007 |
| FRS + GRS2 |  |  |  |  |  |  |
| Cases | 0.125 | 0.0002 | 0.0095 | <0.0001 |  |  |
| Non-cases | 0.0005 | 0.988 | 0 | 0.997 |  |  |
|  | 0.125 | 0.0069 | 0.0095 | <0.0001 | 2.80% | 0.0038 |
| FRS + GRS3 |  |  |  |  |  |  |
| Cases | 0.102 | 0.002 | 0.007 | 0.0002 |  |  |
| Non-cases | 0.005 | 0.875 | 0 | 0.933 |  |  |
|  | 0.107 | 0.015 | 0.007 | 0.0003 | 2.60% | 0.001 |

Table S9 Reclassification results for the comparison of a baseline model including Framingham coefficients and area to a model including genetic risk scores (GRS1, GRS2 and GRS3 respectively) and the baseline model. Genetic risk scores have been derived in all men and validated in men aged 50-59 at baseline).
