# Supplementary material for: Genetic Markers Enhance Coronary Risk Prediction in Men: The MORGAM Prospective Cohorts
Source: PLoS One. 2012 Jul 25;7(7):e40922. doi: 10.1371/journal.pone.0040922 (PMC3405046; doi:10.1371/journal.pone.0040922)
Supplement: Table S10 — Reclassification results comparing a baseline model including family history (FH); to models including genetic risk scores for all men (627 cases, 1342 non-cases). (DOCX) [file pone.0040922.s010.docx]

|  | NRI | | | IDI | |
| --- | --- | --- | --- | --- | --- |
|  | Value | SE | *p* | Value | *p* |
| FRS + FH |  |  |  |  |  |
| Cases | 0.034 | 0.018 | 0.057 | 0.004 | 0.001 |
| Non-cases | -0.020 | 0.015 | 0.193 | 0.001 | 0.416 |
|  | 0.055 | 0.024 | 0.023 | 0.006 | 0.022 |
| FRS + FH + GRS1 |  |  |  |  |  |
| Cases | 0.026 | 0.028 | 0.348 | 0.004 | 0.016 |
| Non-cases | -0.019 | 0.018 | 0.313 | -0.001 | 0.431 |
|  | 0.045 | 0.032 | 0.155 | 0.005 | 0.006 |
| FRS + FH + GRS2 | |  |  |  |  |
| Cases | 0.059 | 0.028 | 0.035 | 0.008 | 0.002 |
| Non-cases | 0.011 | 0.019 | 0.558 | 0.0001 | 0.853 |
|  | 0.047 | 0.033 | 0.147 | 0.008 | 0.003 |

Table S10 Reclassification results comparing a baseline model including family history (FH); to models including genetic risk scores for all men (627 cases, 1342 non-cases).
