# Supplementary material for: Genetic Markers Enhance Coronary Risk Prediction in Men: The MORGAM Prospective Cohorts
Source: PLoS One. 2012 Jul 25;7(7):e40922. doi: 10.1371/journal.pone.0040922 (PMC3405046; doi:10.1371/journal.pone.0040922)
Supplement: Table S11 — Ten year risk of CHD in the population is calculated for a baseline model adjusted for area for 1736 cases and 3082 non-cases related back to the full cohort. Ten year risk with modified risk of each risk factor is calculated by bringing those with above average risk factor values to the population mean and calculating the difference to estimate the proportion of risk attributable to that risk factor. Standard errors and confidence intervals for the statistics were calculated from 2500 bootstrap replications. (DOCX) [file pone.0040922.s011.docx]

| Risk factor | 10 year risk in the population | 10 year risk in the population with modified risk | PAF (95% C.I.) |
| --- | --- | --- | --- |
| GRS1 | 0.082 | 0.072 | 12.1% (SE 0.004) |
| GRS2 | 0.083 | 0.073 | 11.9% (8.8-15.4) |
| GRS3 | 0.083 | 0.075 | 9.5% (5.1-13.6) |
| HDL cholesterol | 0.0809 | 0.071 | 12.3% (9.3-15.7) |
| Systolic BP | 0.081 | 0.071 | 12.1% (8.8-15.4) |
| BMI | 0.081 | 0.076 | 5.9% (3.4-8.5) |
| Diastolic BP | 0.081 | 0.0771 | 4.8% (2.3-7.4) |

Table S11 Ten year risk of CHD in the population is calculated for a baseline model adjusted for area for 1736 cases and 3082 non-cases related back to the full cohort. Ten year risk with modified risk of each risk factor is calculated by bringing those with above average risk factor values to the population mean and calculating the difference to estimate the proportion of risk attributable to that risk factor. Standard errors and confidence intervals for the statistics were calculated from 2500 bootstrap replications.
