# Supplementary material for: Genetic Markers Enhance Coronary Risk Prediction in Men: The MORGAM Prospective Cohorts
Source: PLoS One. 2012 Jul 25;7(7):e40922. doi: 10.1371/journal.pone.0040922 (PMC3405046; doi:10.1371/journal.pone.0040922)
Supplement: Supporting Information S1 — (DOCX) [file pone.0040922.s012.docx]

Supporting Information

10-fold cross validation

In validating the data-derived scores, the possibility of overfitting needs to be taken into account. This may be avoided by ensuring that same individual is never used for both deriving and validating the risk model. While split-sample validation, that is, splitting the data into two disjoint sets for deriving the score and validating it, is conceptually simple, the split into the two sets is arbitrary and typically results in reduced efficiency. Cross validation utilizes the available data much more efficiently, since the risk model is derived and validated based on larger subsets of the data, while still avoiding overfitting. We utilized 10-fold cross validation, where the data was randomly split into 10 equal sized groups. The risks were calculated for each of the 10 groups from a risk model fitted to the data with this group omitted.

Missing genetic data

The level of missing genotype data in the MORGAM case-cohort set either due to unsuccessful genotyping or unavailable specimens ranged from 10.2 to 21.4% (mean 13.1%) per SNP. The Augsburg cohort (cohort 1) has 18 years of follow up with some high levels of missing data (success rate 86% for most SNPs) which is mainly due to the quality and amount of DNA remaining for these individuals. Using only completely genotyped cases in deriving and validating the multivariate genetic risk scores would lose a lot of information, which is why we applied multiple imputation to more efficiently utilize the available data. However, to make the results less sensitive to the imputation model, we restricted the validation set to individuals with complete genotypes available for >11 of 15 SNPs with the remainder imputed. Rather than substituting estimates to missing genotype values, the main objective of multiple imputation is to estimate the uncertainty about the missing values. This method does not use a reference panel of SNPs in LD like imputation programs such as IMPUTE but simply imputes missing values using a Bayesian approach using the information provided by the data. If the missing values are assumed to be missing at random, the missingness may depend on all observed data (but not the missing values themselves) (Little & Rubin 1987). Statistical analysis accounting for this missingness does not bias the inference as long as the dependencies between the variables which may have accounted for the missingness are properly modelled. The technique makes full use of the information provided by the data without creating any artificial information or bias. Imputation of missing values has been shown to be superior to complete case analysis and the missing-indicator method in multivariable diagnostic research (Moons *et al.* 2006).

The MRAS SNP

The rs9818870 SNP representing the MRAS region was selected according to our SNP selection criteria however data on this SNP was not collected for the Swedish cohort. As multiple imputation was performed on a cohort specific basis we did not impute this SNP for this cohort to avoid additional modelling assumptions. However we carried out a sensitivity analysis to explore the effect of adding this SNP to genetic risk score 1 (externally derived score with total 12 SNPs and two haplotypes) but excluding the Swedish cohort. The rs9818870 SNP was weighted in the score with a hazard ratio of 1/1.15, see Table 2. Analysis of all men consisted of 608 cases and 1307 non-cases. The baseline model using the Framingham Score achieved a c-index of 0.739. Adding GRS1 (with rs9818870) to the baseline model did not significantly improve discrimination (c-index 0.749, *p*=0.11). However, risk classification improved significantly (NRI by 7.6% *p*=0.017 and IDI by 0.4% *p*=0.003) in the entire sample of men (Supplementary table 7). Clinical NRI for men in the intermediate risk group only (416 cases, 637 non-cases) was not significant (6%, *p*=0.15). The result based on men aged 50-59 at baseline (424 cases and 581 non-cases). The Framingham score gave a c-index value of 0.658. C-index improvement was 2.4% (*p*=0.014) with corresponding NRI 12.9% (*p*=0.0039), IDI 0.6% (*p*=0.0033). Therefore these results are consistent with results obtained on the larger dataset. Analysis of this dataset without the MRAS SNP and Swedish cohort did not change the results appreciably, results available on request.

Sites and key personnel of contributing MORGAM Centres:

**Finland**

FINRISK, National Institute for Health and Welfare, Helsinki: V. Salomaa (principal investigator), A. Juolevi, E. Vartiainen, P. Jousilahti;

ATBC, National Institute for Health and Welfare, Helsinki: J. Virtamo (principal investigator), H. Kilpeläinen;

MORGAM Data Centre, National Institute for Health and Welfare, Helsinki: K. Kuulasmaa (responsible person), Z. Cepaitis, A. Haukijärvi, B. Joseph, J. Karvanen, S. Kulathinal, M. Niemelä, O. Saarela;

MORGAM Central Laboratory, National Institute for Health and Welfare, Helsinki: L. Peltonen (responsíble person), M. Perola, K. Silander, M. Alanne, P. Laiho, K. Kristiansson, K. Ahonen;

**France**

National Coordinating Centre, National Institute of Health and Medical Research (U258), Paris: P. Ducimetière (national coordinator), A. Bingham;

PRIME/Strasbourg, Department of Epidemiology and Public Health, EA 3430, Faculty of Medicine, University of Strasbourg, Strasbourg: D. Arveiler (principal investigator), B. Haas, A. Wagner;

PRIME/Toulouse, Department of Epidemiology, Toulouse University School of Medicine, Toulouse: J. Ferrières (Principal Investigator), J-B. Ruidavets, V. Bongard, D. Deckers, C. Saulet, S. Barrere;

PRIME/Lille, Department of Epidemiology and Public Health, INSERM U744-Université Lille Nord de France – Institut Pasteur de Lille: P. Amouyel (principal investigator), M. Montaye, B. Lemaire, S. Beauchant, D. Cottel, C. Graux, N. Marecaux, C. Steclebout, S. Szeremeta;

MORGAM Laboratory, INSERM U937, Paris: F. Cambien (responsible person), L. Tiret, V. Nicaud;

**Sweden**

Northern Sweden, Umeå University Hospital, Department of Medicine, Umeå: P-G. Wiklund (principal investigator), B. Stegmayr (former principal investigator), K. Asplund (former principal investigator), S. Nasic, G. Rönnberg, Å. Johansson, V. Lundberg, E. Jägare-Westerberg, T. Messner;

**Germany**

MONICA/KORA Augsburg, Helmholtz Zentrum München, German Research Center for Environmental Health, Neuherberg: H. E. Wichmann (principal investigator), A. Peters, B. Thorand, C. Meisinger, A. Döring, J. Baumert, A. Schneider, T. Illig, N. Klopp, C. Gieger.

MORGAM laboratory, Research Unit of Molecular Epidemiology, Helmholtz Zentrum München, Neuherberg: T. Illig (responsible person), N. Klopp.

**United Kingdom**

PRIME/Belfast, Queen's University Belfast, Belfast, Northern Ireland: F. Kee (principal investigator) A. Evans (former principal investigator), J. Yarnell, E. Gardner;

MORGAM Coordinating Centre, Queen's University Belfast, Belfast, Northern Ireland: A. Evans (MORGAM coordinator), S. Cashman, F Kee;

MORGAM Management Group: A. Evans (chair, Belfast, UK), S. Blankenberg (Hamburg, Germany), F. Cambien (Paris, France), M. Ferrario (Varese, Italy), K. Kuulasmaa (Helsinki, Finland), A. Palotie (Cambridge, UK), M. Perola (Helsinki, Finland), A. Peters (Neuherberg, Germany), V. Salomaa (Helsinki, Finland), H. Tunstall-Pedoe (Dundee, Scotland), P.G. Wiklund (Umeå, Sweden); Previous members: K. Asplund (Stockholm, Sweden), L. Peltonen (Helsinki, Finland), D. Shields (Dublin, Ireland), B. Stegmayr (Umeå, Sweden)

Cardiogenics Management Group: H. Schunkert (chair), J. Erdmann (Executive secretary), N. Samani (Leicester, UK), W. Ouwehand (The Netherlands), C. Hengstenberg (Regensburg, Germany), P. Deloukas (Sanger UK), A. Rice (UK), F. Cambien (Paris, France), A Evans (Belfast UK).
